# Complete chloroplast genome sequences of *Poikilospermum lanceolatum* (Urticeae)

**DOI:** 10.1080/23802359.2019.1623117

**Published:** 2019-07-11

**Authors:** Xianhan Huang, Tao Deng, Nan Lin, Zhenyu Lv, Xu Zhang, Zhuo Zhou, Yuehua Wang, Hang Sun

**Affiliations:** a School of Life Sciences, Yunnan University, Kunming, China;; b Key Laboratory for Plant Diversity and Biogeography of East Asia, Kunming Institute of Botany, Chinese Academy of Sciences, Kunming, China;; c University of Chinese Academy of Sciences, Beijing, China;; d Key Laboratory of Plant Germplasm Enhancement and Specialty Agriculture, Wuhan Botanical Garden, Chinese Academy of Sciences, Wuhan, China;; e School of Life Sciences, Yunnan Normal University, Kunming, China

**Keywords:** Chloroplast genome, *Poikilospermum lanceolatum*, phylogenetic analysis

## Abstract

The complete chloroplast genome of *Poikilospermum lanceolatum* was sequenced using HiSeq4000 of Illumina. The length of this genome was 153, 454 bp, including a large single copy (LSC) region (84,202 bp), a small single copy (SSC) region (18,172 bp), and two inverted repeat (IR) regions (25,540 bp). The overall GC content of the genome of *P. lanceolatum* was 36.9%. The genome included 111 unique genes (78 protein-coding genes, 29 tRNA genes, and four rRNA genes). Phylogenetic analysis based on 67 protein-coding genes showed that Boehmerieae was sister to *P. lanceolatum*, with 100% bootstrap values.

*Poikilospermum* Zippelius ex Miquel (Urticeae) consists of approximately 27 species and is distributed from Sino-Himalayan region through Malaysia to the Bismarck Archipelago (Chew 1963; Chen et al. 2003). This genera was published by Miquel in 1864 (Miquel 1864); however, its systematic position has been controversial since then (Berg 1978; Chen et al. 2003). Up to now, there is no any species of *Poikilospermum*, even Urticeae with reported complete plastid genome. Therefore, we aim to establish and characterize the complete chloroplast genome of *Poikilospermum lanceolatum* and ascertain its phylogenetic position in this study.

Fresh leaves of *P*. *lanceolatum* were collected from Bulangxiang town of Menghai county in Yunnan Province (21°34′43.914″ N, 100°19′59.4624″ E). The voucher specimens were deposited in the Herbarium of Kunming Institute of Botany, Chinese Academy of Sciences (KUN, ZH026). *P*. *lanceolatum* was sequenced using HiSeq4000 of Illumina at the Beijing Novogene Bioinformatics Technology Co., Ltd (Nanjing, China). NOVOPlasty 2.7.2 (Dierckxsens et al. 2017), a seed-extend based assembler, was used to assemble the plastome and *Boehmeria spicata* (NC_036989) was used as the seed sequence. The plastome of *P*. *lanceolatum* was annotated using Geneious 9.0.2 (Kearse et al. 2012) against the plastome of *B. spicata* and *Morus mongolica* (KM491711). The DOGMA was used to correct the annotation (Wyman et al. 2004).

The complete chloroplast genome of *P. lanceolatum* (MK778867) was 153,454 bp in size. This genome was with a typical quadripartite structure, containing SSC with the length of 18,172 bp, LSC with the length of 84,202 bp and two IR regions with the length of 25,540 bp. The GC content of this genome was 36.9%. There were 130 predicted functional genes in the genome, including 111 different genes (78 protein-coding genes, 29 tRNA genes, and four rRNA genes) and 19 duplicated genes. In the 111 genes, 19 included one intron, and only three included two introns (clpP, rps12, and ycf3).

Phylogenetic position of *P. lanceolatum* was analysed based on the 67 shared protein-coding gene sequences of this species and other 11 species, belonging to Boehmerieae of Urticaceae, Cannabaceae, Rosaceae, and Ulmaceae. The sequences were aligned in MAFFT (Katoh et al. 2002). The maximum-likelihood phylogenetic tree was reconstructed using RaxML (Stamatakis 2014). The ML analysis showed that *P. lanceolatum* was sister to Boehmerieae with 100% bootstrap values, which was congruent with the precious studies (Kim et al. 2015; Wu et al. 2013) (Figure 1). Our results here could be further applied for evolutionary and phylogenetic studies of *Poikilospermum*, Urticeae, even Urticaceae.

**Figure 1.**
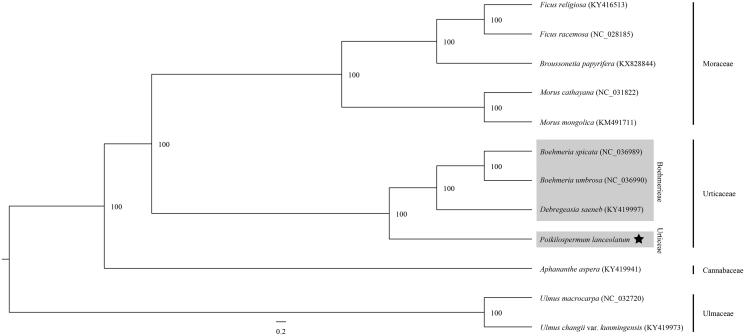
Phylogeny of *Poikilospermum lanceolatum* and other 11 species of Rosales based on the 67 common protein-coding gene sequences using maximum-likelihood method. The numbers at the nodes indicate bootstrap support values.
